# Decreased purine ingestion, increased molybdenum ingestion, and the decline in amyotrophic lateral sclerosis on Guam

**DOI:** 10.3389/fnut.2025.1618834

**Published:** 2025-10-13

**Authors:** Christopher A. Bourke

**Affiliations:** Veterinary Practitioner, Millthorpe, NSW, Australia

**Keywords:** diet, purines, molybdenum, Chamorro, Guam, amyotrophic lateral sclerosis

## Introduction

Amyotrophic lateral sclerosis once affected the Chamorro people of Guam at a very high rate (1). It no longer does. Most agree that the decline occurred because of a dietary change that followed the United States occupation in 1944. Many thought this was the cessation in consumption of cycad flour, which is a product made from the nut of the *Cycas micronesica* palm. Some continue to support this hypothesis (2–6), but just as many do not (7–11). Preoccupied by this ongoing debate, investigators have overlooked other dietary changes that occurred on Guam post-1944. These other changes could equally be responsible for the decline in ALS. Two such changes were: (1) a reduction in the purine content of the Chamorro diet, and (2) an increase in its molybdenum content. There is a link between purines and molybdenum, the enzyme that catabolizes the former has an obligatory requirement for the latter. There is also a link between purines, molybdenum and the development of motor nervous disorders in some animal species (12, 13).

## What is amyotrophic lateral sclerosis

ALS is a motor nervous disorder that is divisible into three parts (14, 15). All three parts occur in humans and cats (16), parts 1 and 2 occur in sheep and part 3 occurs in horses. The three parts are;

(1) Asymmetric, limb, antigravitational muscle weakness, due to dysfunctions of, the upper motor neuron, the ponto-medullary extensor muscles facilitatory center, and the spinal motor neurons (12, 17). This part has been produced in molybdenum deficient sheep fed the purine xanthosine (12).

(2) Bulbo-respiratory muscle weakness, due to dysfunctions of, the upper motor neuron, and the ponto-medullary respiratory muscles facilitatory center (13). This part has been produced in molybdenum deficient sheep fed the purine inosine (13).

(3) Generalized muscle weakness, due to spinal motor neuron degeneration (14, 15). This part occurs in isolation in horses and appears to involve the molybdenum dependant enzyme sulphite oxidase (14, 15), with glutathione deficiency hence vitamin E deficiency a consequence (14, 15).

Human ALS can initially present as either limb antigravitational muscle weakness or bulbo-respiratory muscle weakness, but eventually involves both. Generalized trunk muscle weakness occurs later in the course of the disease in most cases.

## The timeline for the decline in ALS on Guam

From 1945–49 the average annual incidence of ALS in Chamorro men was 67 per 100,000 and in women 37 (18). By 1975–79 it had dropped to 17 per 100,000 for men and 12 for women (18). By 1995–99 it was 1.0 per 100,000 for men and 2.0 for women (18). Assuming a dietary cause, the initial sex difference in incidence would suggest that the food choices of men on the traditional diet were different to those of women. However, after 64 years of the American diet, there was no longer any identifiable sex difference in food choices (19), nor in the incidence of ALS. By subtracting the mean 14 years long preclinical latent period for ALS (20), the proposed dietary change responsible for the ALS decline can be seen to have occurred, for the majority of Chamorro, by 16 years post-1944. Part of the observed decline in disease incidence could be attributable to migration, improved survival, changes in diagnostic criteria and competing mortality. In addition, the possible contribution made by genetic dilution and environmental toxins cannot be excluded.

## The traditional Chamorro diet prior to 1944

The stated Chamorro diet prior to 1944 is retrospective hence reliant on assumptions. There was an oral history pre-1944 but no documented records. What we do know is that before 1944 all food consumed by the Chamorro was grown or gathered on the island (21–24). It was a subsistence diet heavily based upon plant foods. Fish and seafoods were also wild harvested and commonly eaten (22–24). Meat was not a common food source for the Chamorro, but chickens, pigs and flying foxes (bats) were eaten by some on special occasions. The foods that were grown in village food gardens were; taro, yams, seedless breadfruit, rice, arrowroot, sweet potato, cassava, corn (maize), sugar cane, ginger, melons, and leguminous beans. The wild foods that were gathered were; bananas, seeded breadfruit, coconuts, cycad nuts, giant taro, and wild yams. The main protein sources for the Chamorro were fish and seafoods harvested along the coastline of Guam and eaten and traded between coastal and inland villages. The seafoods included; clams, sea urchins, shrimps, lobster, crabs, crayfish, octopus, and oysters.

## The American diet eaten by the Chamorro from 1945 onwards

After 1944, U.S. imports of food replaced the traditional foods of the Chamorro. The rapid development of a cash economy resulted in a move away from growing and gathering food to buying it in supermarkets. The American diet was heavily based on processed rice, refined wheat flour, potatoes, sugar, chicken, pork, beef, and sausages (19). By 2008 the Chamorro diet included the following foods; processed white rice, sweetened beverages, spaghetti & meat sauce, French fries, beer, chorizo sausage, tacos, red rice, Kadun chicken stew, orange juice, McDonalds crispy chicken sandwich, fried rice, Adobo chicken stew, hotdogs, white bread, pork spareribs, sweetened cereals, beef pot roast, chop-steak, sweet tortillas, scrambled eggs, ice cream, sugar, milk, and donuts (19).

## The purine status of the traditional Chamorro diet and the American diet

The purine status of the traditional Chamorro diet was never measured, hence values presented here are assumed estimates based upon our current knowledge of the purine content of individual food stuffs today. The purines of interest for ALS, are hypoxanthine (hence inosine), and xanthine (hence xanthosine). Their content for a range of present-day foods is presented in Table 1 (25). In plant-based foods hypoxanthine and xanthine are very low. For the former less than 2.4 mg per 100 g, and for the latter less than 0.6 mg per 100 g. The meat-based foods, together with fish and seafoods (except crab), are all good sources of hypoxanthine. With the average hypoxanthine content for meats being 51 mg per 100 g, for fish 95 mg and for seafoods 35 mg. The amount of xanthine in most meat-based foods is small, only 2.3 mg per 100 g. Beef is the exception, being 11.8 mg per 100 g. Fish contains very little xanthine, less than 1 mg per 100 g, but seafood collectively averages 50 mg per 100 g.

**Table 1:** The hypoxanthine and xanthine content of a range of foods (24).

**Foodstuff**	**Hypoxanthine, mg/100 g**	**Xanthine, mg/100 g**
Unpolished rice	Nil	Nil
Sweet potato	2.4	0.6
Taro	0.2	Nil
Yam	0.2	Nil
Bananas	0.1	Nil
Broad beans	1.3	0.2
Cucumber	0.1	0.1
Ginger	Nil	0.5
Polished rice	Nil	Nil
Corn/maize	0.1	0.1
Potatoes	0.2	Nil
Wheat flour	Nil	Nil
Fish (mean 7 species)	94.6	0.9
Squid	34.0	53.2
Lobster	61.2	0.1
Clams	12.2	30.4
Sea urchins	22.5	9.1
Shrimps	103.4	117.1
Crabs	Nil	47.2
Oyster	12.2	82.1
Octopus	36.7	60.8
Canned corn beef	31.3	1.5
Canned ham meat	55.8	Nil
Vienna sausage	32.7	Nil
Frankfurt sausage	32.7	1.5
Chicken (mean 4 different cuts)	82.2	1.4
Pork (mean 6 different cuts)	63.0	Nil
Beef (mean 10 different cuts)	57.6	11.8
Eggs (chicken)	Nil	Nil
Milk	Nil	Nil
Cheese	Nil	Nil

The fish and seafood components of the traditional Chamorro diet presumably made it rich in both hypoxanthine and xanthine. By contrast the contribution of chicken, pork and beef meats to the American diet provided only a moderate amount of hypoxanthine and very little xanthine. This difference would have been further exaggerated by the American diet being low in fiber, and high in sugar, this would have encouraged greater consumption of plant-based foods relative to meats.

In Mo deficient sheep the ingestion of xanthosine at the rate of 14 mg/kg body weight per day for a period of 32 weeks (12), produced delayed onset, irreversible, asymmetric limb antigravitational muscle weakness approximately 30 months afterwards. Likewise, feeding inosine, at a slightly higher rate, produced delayed onset, irreversible, bulbar and respiratory muscle weakness approximately 22 months later (13).

## The Mo status of the traditional Chamorro diet and the American diet

Neither the Mo status of the traditional diet, nor the present-day American diet, on Guam, have ever been measured. However, the Mo status of the diet being eaten by Americans in the U.S. in 1980 has been determined. The Mo content of the daily average diet consumed in the U.S. was 0.07 ppm (15, 26). The American diet on Guam would have moved toward this value from 1945 onwards. A reasonable determination of plant food Mo content on Guam prior to 1945 can be made by using what we known of the geology of Guam, the soil pH values established in 1963 (27), and the soil Mo values established in 2002 (28, 29). This process is of necessity based upon assumptions, hence the Mo values determined are not precise.

**Figure 1:**
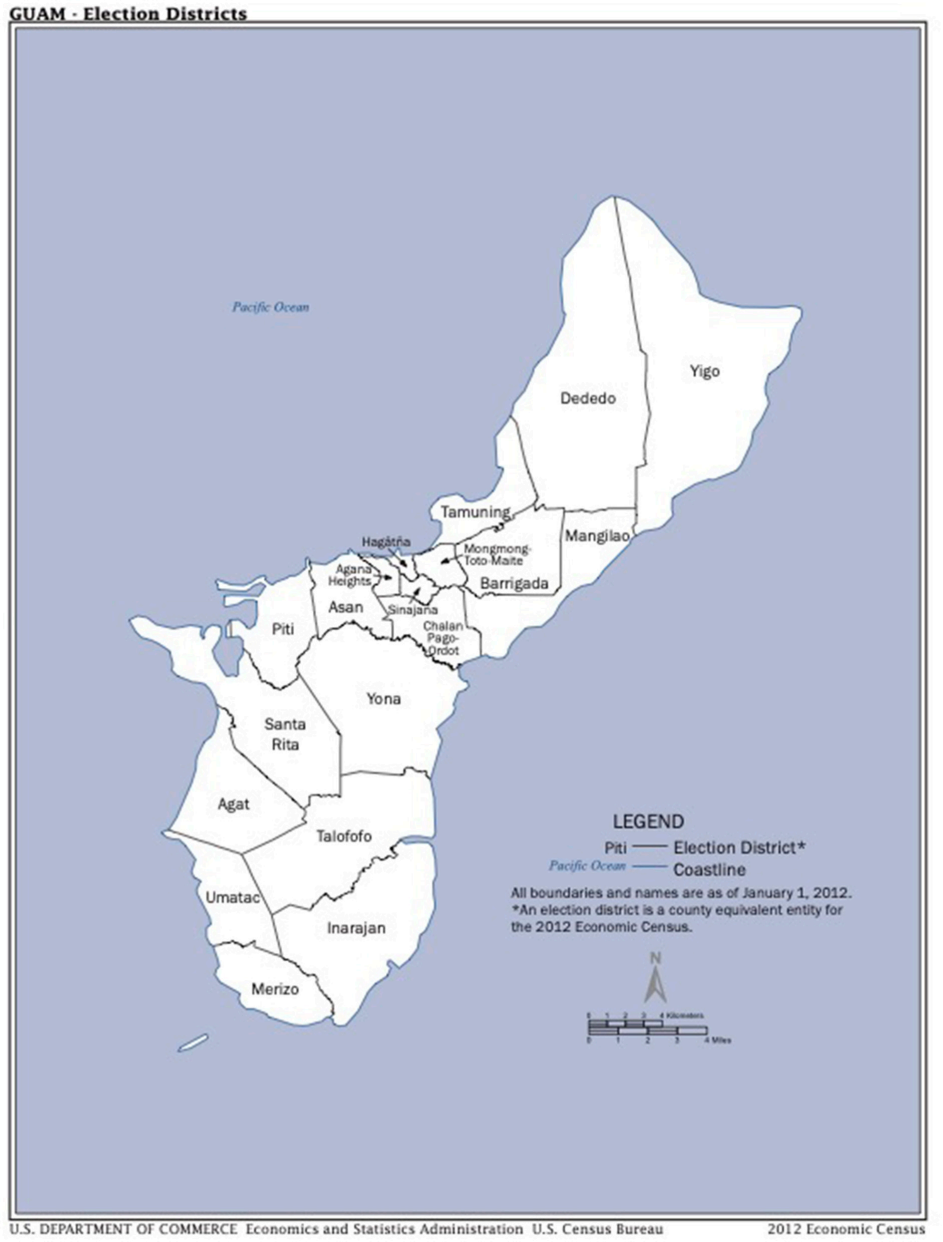
The election districts of Guam.

Guam is divisible into roughly equal northern and southern parts (see Figure 1). In the north, the soils are neutral to alkaline limestone soils derived from carbonate rocks. In the south, the soils are acidic volcanic soils derived from volcanic rocks (28, 29). This much is certain because the geology of the island has been stable for thousands of years. Soil Mo availability in limestone soils is moderate, and in acidic volcanic soils low. Unlike most metal nutrients Mo is much less bioavailable under acidic conditions. Mo is recalcitrant at low pH because of the ligand exchange mechanism, which results in Mo being bound to soil particles hence not available to plants (30). In addition, Mo is strongly fixed in soil by oxides of iron, aluminum and manganese. The pH values established for Guam's volcanic soils in 1963 ranged from pH 5.1–6.8 (mean 5.6), whereas those for the limestone soils ranged from pH 6.1–7.8 (mean 7.1) (27). The relative availability of Mo at pH 7.0 is double that available at pH 5.5 (31). Food crops grown on these two soil types would have reflected this difference in Mo availability. However, caution is required because of the absence of historical soil pH data.

When the Mo content of the acidic volcanic soils was established for the first time in 2002 the following values were obtained (28, 29). For 13 samples collected in Umatac, the mean was 0.86 ppm (range 0.1–1.3). Four samples from Merizo were 1.0 ppm (0.7–1.6), 3 from Inarajan were 1.2 ppm (1.1–1.3), and 4 from Santa Rita were 0.43 ppm (0.2–0.7). A single sample was collected from the limestone soils of Dededo in northern Guam. It had a Mo value of 4.3 ppm. The southern samples gave consistently low Mo values whereas the random sample from the north had a moderate Mo value, as would be expected. Historical soil Mo values will never be known and therefore the relative Mo content of food crops grown on Guam in past decades can only be assumed.

Mo is required for the growth of plants such as beans, peas, peanuts and cycads, the roots of which fix nitrogen. It is not required for the growth of other plants. That said, all plants will pick up whatever Mo is available in the soil. Consequently, the same plant species can have either a very high or a very low Mo content, depending on the soil in which its grown (12). Annual crop growth and crop removal, without mineral replacement, gradually reduces soil Mo availability hence plant Mo content. For example, in Australia after 60 years of crop growth and crop removal on acidic volcanic soils (12), the plant Mo content fell to < 0.01 ppm. The traditional Chamorro diet, in the southern election districts, where food had been grown on acidic volcanic soils for centuries, could conceivably have dropped to this same low level.

The incidence of ALS in the southern villages was known to be much higher than that in the northern villages. Election district data is not available for ALS but it is available for Parkinsonism-Dementia complex, the disease that occurred together with ALS and for which a purine etiology has previously been proposed (32). The district by district, average annual incidence of PDC in Chamorro men, during the period 1956–1965, was 85 per 100,000 for the southern districts but only 47 for the northern districts (33). This almost 2 fold difference matches the assumed background difference in soil pH, hence soil Mo availability, between these two zones.

## Discussion

There has been an overriding assumption that Mo deficiency very rarely causes clinical disease in humans. Only one case is cited in the literature (34). This was an adult male with Crohn's disease on parenteral nutrition for 18 months. The biochemistry was as expected for Mo deficiency, namely depressed urates, increased plasma methionine, depressed urine sulfates but increased urine thiosulphates. These changes were consistent with very low xanthine oxido-reductase and sulfite oxidase enzyme activity. It was concluded that the clinical disease observed was attributable to sulfur amino acid intolerance and that the cause was Mo deficiency. None of the historical animal studies into the potential effects of Mo deprivation have included either a dietary purine challenge or a dietary sulfur amino acid challenge, in their protocols. Without either of these no clinical effect was achieved nor should it have been expected. Likewise, the animal species selected for these studies were not purine salvaging species, for any purine challenge or monogastric species, for any sulfur amino acid challenge. Because of these short comings clinical disease in experimental animals had not been demonstrated until Bourke in 2012 (12).

Four purine nucleosides and their corresponding nitrogenous bases occur in foods. They are xanthosine (xanthine), inosine (hypoxanthine), adenosine (adenine), and guanosine (guanine). Each nucleoside contains a ribose sugar but its corresponding base does not. Because purines normally occur in the central nervous system, the body must protect itself from dietary sources of them. There are two levels of protection. The first is the enzyme xanthine oxido-reductase, located in the gut wall and liver, which acts by catabolizing purines. This enzyme has an absolute requirement for Mo. The second is a capacity shared by all animal species whereby they can excrete surplus un-catabolized purines. Humans, sheep, goats, cats, and pigs, are different to all other animals in that they have the ability to salvage un-catabolized purines, this ability is associated with an absence of serum XOR (35). This capacity for salvage is both a potential benefit and a potential risk. When XOR enzyme activity is too low, humans and sheep inadvertently load salvaged purines into their CNS (12, 13). This outcome is potentially neurotoxic. Consequently, Mo deficiency in humans and sheep can lead to dire neurological consequences. Animal studies have shown that when the purines xanthosine and inosine are eaten by Mo deficient sheep, they develop the majority of clinical signs that characterize ALS in humans (12, 13). This purine plus Mo deficiency association with human ALS is supported by the frequent finding of low blood urate levels in patients with ALS (36). Low blood urate levels are indicative of low xanthine oxidase activity, and low xanthine oxidase activity is caused by Mo deficiency. The purine plus Mo deficiency association with ALS is further supported by the finding that the prevalence of ALS is significantly lower in gout patients when compared to the general population (37). Gout is caused by elevated dietary purine intakes in persons with very good XOR activity hence a strong Mo status. Plasma cysteine is increased in ALS and urine sulfates decreased (38). This is also consistent with Mo deficiency. As is the finding that serum levels of homocysteine are increased in ALS (39). There is evidence to suggest that some sporadic cases of ALS are in part due to genetic mutations (40). Consequently, it is possible that Mo deficiency is the environmental factor that leads to ALS in individuals of a particular genotype.

Some may query the relevance to humans of findings in animals such as sheep. In this regard it should be remember that *Homo sapiens* is also an animal, evolved from the great apes. Sheep and humans share a similar body size, a similar mid-brain and brain stem size, and identical purine salvage physiology. Humans have a large and highly developed cerebral cortex, including the motor cortex and a fully functional cortico-spinal tract (pyramidal tract). These evolutionary developments give humans greater powers of memory, cognition, imagination and fine motor skills. Sheep have a smaller cerebral cortex, a simple motor cortex and a vestigial cortical-spinal tract. However, in the midbrain and brain stem area sheep share the same anatomical size and the same motor functional ability as humans. This ability centers around the constant automatic control of limb antigravitational muscle and bulbo-respiratory muscle activation. These two involuntary functions keep humans and sheep upright when awake, allow them to vocalize and swallow, and ensure that they continue to breath even when in a resting or sleeping state. These auto-controls are the underlying functions that fail in ALS. In these respects the sheep is a perfect match for the human.

ALS continues to occur on Guam. This is an important fact, as is the observation that ALS on Guam now has a similar incidence rate to that of ALS in the U.S., being 1.5 per 100,000 on Guam and 1.8 in the U.S. (18, 41). This equalization supports a dietary cause that is common to both countries. The continuation of ALS on Guam, albeit at a more normal incidence, and the identical clinical presentation of it on Guam to that of ALS in the U.S. confirms that they are one and the same disease and that they have the same cause. The causal, presumably dietary, factor on Guam has not gone away it has just declined in its significance. The decline in the incidence of ALS on Guam coincided with a decline in the amount of xanthosine and inosine present in the Chamorro diet, together with an apparent, significant increase in the Mo content of that diet. Two components of diet that are shared by Guam and the U.S. are purines and molybdenum. Assuming that the Mo content of the diet on Guam in 1944 was as low as < 0.01 ppm, and assuming that by 1980 the American diet had lifted it to 0.07 ppm (15, 26), this and a reduced purine intake would explain the current parity in ALS incidence between the two countries. Naturally occurring field outbreaks of a profound, chronically progressive, irreversible, asymmetric, limb, antigravitational muscle weakness, occur in xanthosine ingesting sheep when their dietary Mo level falls below 0.04 ppm (12).

## References

[1] Morris HR Al-Sarraj S Schwab C Gwinn-Hardy K Perez-Tur J Wood NW. A clinical and pathological study of motor neurone disease on Guam. Brain. (2001) 124:2215–22. 10.1093/brain/124.11.2215 11673323

[2] Spencer PS Nunn PB Hugon J Ludolph AC Ross SM Roy DN. Guam amyotrophic lateral sclerosis-parkinsonism-dementia linked to a plant excitant neurotoxin. Science. (1987) 237:517–22. 10.1126/science.3603037 3603037

[3] Ince PG Codd GA. Return of the cycad hypothesis – does the amyotrophic lateral sclerosis/parkinsonism dementia complex of Guam have new implications for global health. Neuropathol Appl Neurobiol. (2005) 31:345–53. 10.1111/j.1365-2990.2005.00686.x 16008818

[4] Cox PA Banack S Murch S Sacks O. Commentary on: return of the cycad hypothesis. Neuropathol Appl Neurobiol. (2006) 32:679–82. 10.1111/j.1365-2990.2006.00796.x 17083482

[5] Borenstein AR Mortimer JA Schellenberg GD Glasko D. The ALS/PDC syndrome of Guam and the cycad hypothesis. Neurology. (2009) 72:473–6. 10.1212/01.wnl.0000344257.59693.cf 19188582

[6] Rivadeneyra-Dominguez E Rodriguez-Landa JF. Cycads and their association with certain neurodegenerative diseases. Neurologia. (2014) 29:517–22. 10.1016/j.nrleng.2013.03.005 23725821

[7] Steele JC McGeer PL. The ALS/PDC syndrome of Guam and the cycad hypothesis. Neurology. (2008) 70:1984–90. 10.1212/01.wnl.0000312571.81091.26 18490618

[8] Snyder LR Marler TE. Rethinking cycad metabolite research. Commun Integrat Biol. (2011) 4:1:86–8. 10.4161/cib.14084 21509189 PMC3073281

[9] Chernoff N Hill DJ Diggs DL Faison BD Francis BM Lang JR. A critical review of the postulated role of the non-essential amino acid, BMAA, in neurodegenerative disease in humans. J Toxicol Environ Health. (2017) 20:183–229. 10.1080/10937404.2017.1297592 28598725 PMC6503681

[10] Kokubo Y Morimoto S Yoshida M. Questioning the cycad theory of Kii ALS-PDC causation. Nat Rev Neurol. (2024) 20:194. 10.1038/s41582-024-00936-0 38336910

[11] Bourke CA. An eLetter on the *Science* web site (2025), challenging the validity of Spencer et al. Science (1987) 237:517–22.3603037

[12] Bourke CA. Motor neuron disease in molybdenum-deficient sheep fed the endogenous purine xanthosine: a possible mechanism for Tribulus staggers. Aust Vet J. (2012) 90:272–74. 10.1111/j.1751-0813.2012.00947.x 22731949

[13] Bourke CA. Molybdenum deprivation, purine ingestion and an astrocyte-associated motor neurone syndrome in sheep: the assumed clinical effects of inosine. Aust Vet J. (2015) 93:79–83. 10.1111/avj.12286 25708791

[14] Bourke CA. Inosine supplements only reach the CNS in molybdenum deficient humans and may cause astrocyte degeneration and bulbar-respiratory disease. ALS &FD. (2022) 23:154–6. 10.1080/21678421.2021.1947322 34251948

[15] Bourke CA. Molybdenum deficiency produces motor nervous effects that are consistent with amyotrophic lateral sclerosis. Front Neurol. (2016) 7:28. 10.3389/fneur.2016.00028 27014182 PMC4782119

[16] Shelton GD Hopkins AL Ginn PE De Lahunta A Cummings JF Berryman FC. Adult-onset motor neuron disease in three cats. JAVMA. (1998) 212:1271–75. 10.2460/javma.1998.212.08.1271 9569168

[17] Bourke CA. Abnormal turning behaviour, GABAergic inhibition and the degeneration of astrocytes in ovine *Tribulus terrestris* motor neuron disease. Aust Vet J. (2006) 84:53–8. 10.1111/j.1751-0813.2006.tb13128.x 16498837

[18] Plato CC Garruto RM Galasko D Craig UK Plato M Gamst A. Amyotrophic lateral sclerosis and Parkinsonism-dementia complex of Guam: changing incidence rates during the past 60 years. Am J Epidemiol. (2003) 157:149–57. 10.1093/aje/kwf175 12522022

[19] Leon-Guerrero RT Paulino YC Novotny R Murphy SP. Diet and obesity among Chamorro and Filipino adults on Guam. Asia Pac J Clin Nutr. (2008) 17:216–22. 18586639 PMC2762033

[20] Garruto RM Gajdusek DC Chen K-M. Amyotrophic lateral sclerosis among Chamorro migrants from Guam. Ann Neurol. (1980) 8:612–9. 10.1002/ana.410080612 7212649

[21] Pollock N. Food habits in Guam over 500 years. Pac Viewp. (1986) 27:120–43. 10.1111/apv.272002

[22] Salas MC Tolentino D. Digesting the Diets of Guam's ancient Chamorro. Mangilao: Guampedia (2022).

[23] Calleja E. The Ancient Chamorro Diet. Globalization of Food and Health on Guam. Barrigada: Eileen Laureano Calleja (2014).

[24] Ward L.A. The cephalopods of Guam. Micronesica (2003) 35, 36:294–302.

[25] Kaneko K Aoyagi Y Fukuuchi T Inazawa K Yamaoka N. Total purine and purine base content of common foodstuffs for facilitating nutritional therapy for gout and hyperuricemia. Biol Pharm Bull. (2014) 37:709–21. 10.1248/bpb.b13-00967 24553148

[26] Tsongas TA Meglen RR Walravens PA Chappell WR. Molybdenum in the diet: an estimate of average daily intake in the United States. Am J Clin Nutr. (1980) 33:1103–07. 10.1093/ajcn/33.5.1103 7369160

[27] Carroll D Hathaway JC. Mineralogy of Selected Soils From Guam. Geological Survey Professional Paper 403-F. Washington, DC: United States Department of the Interior (1963).

[28] Miller WR Sanzolone RF Lamothe PJ Ziegler TL. Chemical Analyses of Soils, Soil Leaches, Rocks, and Stream Sediments from Guam and the Western United States and Sample Location Maps of Guam. Report 02- 399. Denver, CO: United States Geological Survey (2002). 10.3133/ofr02399

[29] Miller WR Sanzolone RF. Investigation of the Possible Connection of Rock and Soil Geochemistry to the Occurrence of High Rates of Neurodegenerative Diseases on Guam and a Hypothesis for the Cause of the Diseases. Report 03-126. Denver, CO: United States Geological Survey (2003). 10.3133/ofr03126

[30] Duval BD Natali SM Hungate BA. What constitutes plant-available molybdenum in sandy acidic soils? Commun Soil Sci Plant Anal. (2015) 46:318–26. 10.1080/00103624.2014.969405

[31] Underwood EJ Suttle NF. Mineral Nutrition of Livestock. 3rd ed. Ch. 2, Oxford: CABI Publishing (1999). 10.1079/9780851991283.0000

[32] Bourke CA. Astrocyte dysfunction following molybdenum-associated purine loading could initiate Parkinson's disease with dementia. NPJ Parkinsons Dis. (2018) 4:7. 10.1038/s41531-018-0045-5 29581999 PMC5861100

[33] Zhang Z Anderson DW Mantel N. Geographic patterns of Parkinsonism-dementia complex on Guam 1956 through 1985. Arch Neurol. (1990) 47:1069–74. 10.1001/archneur.1990.00530100031010 2222238

[34] Abumrad NN Schneider AJ Steel D Rogers LS. Amino acid intolerance during prolonged total parenteral nutrition reversed by molybdate therapy. Am J Clin Nutr. (1981) 34:2551–59. 10.1093/ajcn/34.11.2551 6795919

[35] Al-Khalidi UAS Chaglassian TH. The species distribution of xanthine oxidase. Biochem J. (1965) 97:318–20. 10.1042/bj0970318 16749120 PMC1264577

[36] Keizman D Ish-Shalom M Berliner S Maimon N Vered Y Artamonov I. Low uric acid levels in serum of patients with ALS: further evidence for oxidative stress? J Neurol Sci. (2009) 285:95–9. 10.1016/j.jns.2009.06.002 19552925

[37] Kwon HS Park Y Kim JH Kim SH Jun J Park S. Prevalence of motor neuron disease in gout patients: a nationwide population-based cohort study. Neurol Sci. (2023) 44:593–600. 10.1007/s10072-022-06451-8 36271260

[38] Heafield MT Fearn S Steventon GB Waring RH Williams AC Sturman SG. Plasma cysteine and sulphate levels in patients with motor neurone, Parkinson's and Alzheimer's disease. Neurosci Let. (1990) 110:216–20. 10.1016/0304-3940(90)90814-P 2325885

[39] Levin J Botzel K Giese A Vogeser M Lorenzl S. Elevated levels of methylmaloate and homocysteine in Parkinson' disease, Supranuclear Palsy and Amyotrophic Lateral Sclerosis. Dement Geriatr Cogn Disord. (2010) 29:553–59. 10.1159/000314841 20606437

[40] Schymick JC Talbot K Traynor BJ. Genetics of sporadic amyotrophic lateral sclerosis. Hum Mol Genet. (2007) 16:R233–42. 10.1093/hmg/ddm215 17911166

[41] Valle J Roberts E Paulukonis S Collins N English P Kaye W. Epidemiology and surveillance of amyotrophic lateral sclerosis in two large metropolitan areas in California. ALS & FD. (2015) 16:209–15. 10.3109/21678421.2015.1019516 25822003 PMC4544858

